# Chronic activation of cardiac Atg-5 and pancreatic Atg-7 by intermittent fasting alleviates acute myocardial infarction in old rats

**DOI:** 10.1186/s43044-022-00268-8

**Published:** 2022-04-13

**Authors:** Sahar Mohamed El Agaty, Noha A. Nassef, Doaa A. Abou-Bakr, Aya A. Hanafy

**Affiliations:** Department of Physiology, Medical Research Center, Faculty of Medicine, Ain Shams University, 24 Mohamed El Makaref Street, Nasr City, Cairo, Egypt

**Keywords:** Aging, Autophagy, Diabetes mellitus, Myocardial infarction, Obesity

## Abstract

**Background:**

Aging is associated with cardiovascular and metabolic changes, increasing the susceptibility to acute myocardial infarction (AMI). Intermittent fasting (IF) has a beneficial effect on the age-associated cardiovascular diseases. The present study was planned to investigate the possible protective effect of IF against acute AMI induced by isoproterenol (ISO) in old rats and its possible underlying mechanisms mediated by heart and pancreatic autophagy. Thirty Male Wistar rats were divided into four groups: adult; old; Old-ISO (rats subjected to AMI by ISO) and Old-F-ISO groups (rats were subjected to IF for 4 weeks and AMI by ISO).

**Results:**

IF significantly increased the mRNA expression of cardiac Atg-5 and pancreatic Atg-7 in Old-F-ISO versus old and adult groups. This was associated with a significant decrease in serum troponin-I, serum creatine kinase (CK-MB), cardiac malondialdehyde and cardiac TNF-α, fasting plasma glucose, and HOMA-IR in Old-F-ISO compared to Old-ISO group. Also, IF significantly decreased the age-related overall and visceral obesity in Old-F-ISO versus old and Old-ISO groups. Histological studies revealed attenuation of the local inflammatory response in Old-F-ISO versus Old-ISO group. Pancreatic Atg-7 and heart Atg-5 were significantly increased in Old-ISO versus old rats.

**Conclusions:**

IF protects against acute AMI in old rats, possibly, via chronic activation of heart Atg-5 and pancreatic Atg-7, and alleviation of age-related overall and visceral obesity. Thus, IF could be a dietary lifestyle modification for attenuation of the susceptibility to acute AMI in aged population. On the other hand, acute activation of heart and pancreatic autophagy by ISO might augment cardiac injury.

## Background

Aging is characterized by slow and progressive structural and functional changes of the heart and blood vessels, leading to cardiac hypertrophy, endothelial dysfunction, coronary insufficiency, and ischemic changes, increasing the susceptibility of old individuals to develop myocardial infarction [1]. Several cardiovascular risk factors have been proposed to mediate the pathogenesis of such detrimental changes. Metabolic comorbidities including obesity, dyslipidemia, hypertension, and hyperglycemia often cluster together in elderly people, promoting the vascular remodeling, endothelial injury and enhancing coronary insufficiency [2]. Moreover, inflammation and oxidative stress are gradually recognized as strong contributors to myocardial infarction with bad prognosis in aged individuals [3, 4].

Autophagy is an evolutionary process that degrades long lived or damaged cellular organelles and proteins for detoxification, energy production, and cellular renewal [5]. Normal basal autophagy is essential for metabolic fitness and adaptation to stressful conditions, such as nutrient deprivation, hypoxia, or oxidative stress [6, 7]. Regulation of mitochondrial autophagy plays a key role in cardiac homeostasis [8]. Activation of autophagy during ischemic injury or ischemia/reperfusion injury was found able to protect cardiomyocytes [9]. Additionally, autophagy was postulated to prevent the inflammatory and oxidative changes, thus attenuating the progression of cardiac injury [10]. Accumulating line of evidence indicates that autophagy declines with aging and such decline initiates age-related cardiovascular diseases, metabolic disorders, and diabetic changes [5]. Disturbed autophagy was postulated to trigger the development of metabolic stresses including obesity, insulin resistance and dyslipidemia in the aging process [11, 12]. Thus, activation of autophagy in aged population might be a potential new strategy that alleviates the age-associated cardiac and metabolic changes which further attenuates the susceptibility to acute cardiac insult.

Intermittent fasting (IF), alternating cycles of fasting and eating, is one of dietary regimens that have been established to increase life expectancy and to reduce the incidence of age-associated diseases, including cancer, diabetes, and kidney disease in animal models [13]. Previous studies had proved that IF can prevent metabolic derangement by reducing body weight [14]; and by improving insulin resistance [15]. Moreover, prolonged IF in human has been shown to produce positive effects on the inflammatory status of the body via decreasing serum levels of IL-6, homocysteine, and C-reactive protein [16]. IF was also reported to attenuate free-radical production and improve cellular stress response [17]. Recently, it has been reported that IF can activate cardiac autophagy and reverse an advanced form of cardiomyopathy [18]. The present study was planned to elucidate the effect of IF on aged heart, and its potential to attenuate acute myocardial infarction, induced by isoproterenol injection. The possible underlying mechanisms mediated by heart and pancreatic autophagy were also investigated. Isoproterenol (ISO) was used to induce AMI in the current work. ISO-induced AMI is a common, reproducible experimental model, producing biochemical, cardiac, and histopathological changes which mimic those of humans [19].

## Methods

### Experimental protocol

This study was carried out on 30 male Wistar rats (10 adult rats, aged 6 month and weighing 170–250 g; and 20 old rats, aged 24 month and weighing 255–450 g). Rats were maintained under regular 12 h:12 h day/night cycle, fed standard rat chow and had free access to water. The animals were acclimatized for 7 days prior to the commencement of the study. All experimental procedures were carried out according to the Guide for the Care and Use of Laboratory Animals published by the US National Institute of Health (No. FWA 00017585; FMASU MS 132/2020).

### Intermittent fasting (IF)

Rats subjected to alternate day fasting were fed ad libitum every other day and fasted the following day with free access to water for 4 weeks. On the fasting day, the cages were changed to avoid the presence of remaining pellets.

### Induction of experimental myocardial infarction

Isoproterenol (Sigma-Aldrich, St. Louis, MO) was freshly prepared in normal saline and injected at a dose of 85 mg/kg body weight by subcutaneous route for two days at 24 h interval (on day 27th and 28th) to induce myocardial infarction [20].

### Experimental design

Rats were randomly assigned into four groups. Adult group (*n* = 10), old group (*n* = 6), old-isoproterenol group, (Old-ISO, *n* = 7); and old-intermittent fasting-isoproterenol group, (Old-F-ISO, *n* = 7). Rats in adult, old, and Old-ISO groups were allowed free access to food and water throughout the experiment. Rats in Old-F-ISO group were subjected to IF. Rats in adult and old groups were injected subcutaneously on day 27th and 28th with normal saline. Rats in Old-ISO and Old-F-ISO groups were subjected to AMI. Anthropometric parameters of all rats were measured at the beginning and at the end of the study. At the end of the experimental period, overnight fasted rats were weighed and anesthetized by intra-peritoneal injection of pentobarbital at a dose of 40 mg/kg. A midline abdominal incision was made, and abdominal aorta was exposed and cannulated with a catheter. Blood samples were collected in EDTA tubes, centrifuged at 5000 rpm for 15 min. The plasma was collected in aliquots and stored at − 80 °C, till used for determination of *glucose homeostasis parameters*, fasting plasma glucose (FPG) and fasting insulin (FI); and *cardiac injury markers*, creatine kinase-myocardial band (CK-MB) and troponin-I (cTnI). Then, the visceral adipose tissue, left ventricle and pancreas were carefully excised. The weight of visceral adipose tissue was determined. Then, a small part of the left ventricle and the pancreas were stored at − 80 °C and used for later determination of cardiac tissue levels of malondialdehyde (MDA), reduced glutathione (GSH), and tumor necrosis factor-alpha (TNF-α); and the mRNA expression of cardiac autophagy related-5 (Atg-5), as well as pancreatic autophagy related-7 (Atg-7). Then, the remaining part of the left ventricles were preserved in 10% formalin for histopathological examination.

### Biochemical analysis

#### Measurement of glucose homeostasis parameters

FPG was assayed by using kits supplied by Diamond Diagnostics, Egypt [21]. FI was measured by a quantitative sandwich enzyme immunoassay (ELISA) technique, using kits supplied by My BioSource company, USA.

Homeostasis Model Assessment of Insulin Resistance (HOMA-IR) and Beta Cell Function (HOMA-%B) were calculated according to [22], as follows:

- HOMA-IR = [Fasting insulin (mU/l) × Fasting glucose (mg/dl) × 0.0555]/22.5
- HOMA-%B = [20 × Fasting Insulin (mU/l)]/[(Fasting Glucose (mg/dl) × 0.0555) − 3.5]

#### Measurement of cardiac injury markers, CK-MB and Troponin-I

CK-MB and cTnI were quantitatively determined in plasma using kits supplied by BioMed Diagnostics company, Egypt [23], and rat specific enzyme immunoassay (ELISA) kits supplied by My BioSource company, USA, respectively.

#### Measurement of cardiac tissue levels of oxidative stress markers, MDA and GSH

MDA and GSH were determined using kits supplied by Biodiagnostic, Egypt.

#### Measurement of cardiac tissue level of proinflammatory marker, TNF-α

TNF-α was measured quantitatively by using rat specific TNF-α immunoassay (ELISA) kits supplied by Cusabio company, USA.

### Determination of the autophagy markers in cardiac tissue (Atg-5) and pancreatic tissue (Atg-7) by quantitative real-time PCR

Total RNA was isolated using Qiagen kit, USA according to instructions of manufacture. The total RNA was used for cDNA conversion using high capacity cDNA reverse transcription kit Fermentas, USA. Real-time qPCR amplification and analysis were performed using an Applied Biosystem with software version 3.1 (StepOne™, USA). The qPCR assay with the primer sets was optimized at the annealing temperature. The used Atg-5 forward primer was 5′-ACGATGACCTGTGTCGAACT-3′, and the reverse primer was 5′-AAACCAAATCTCACTAACATCTTCT-3′. The Atg-7 forward primer was 5′-GAGAGTACATCCCCACCGTC-3′, the reverse primer was 5′-AGGGATCGTACACACCGACT-3′. Beta actin was used as a control housekeeping gene, its forward primer was 5′-TGTTTGAGACCTTCAACACC-3′, the reverse primer was 5′-CGCTCATTGCCGATAGTGAT-3′. The relative quantitation was calculated according to Applied Bio system software.

### Histological examination

The left ventricles were fixed in 10% formalin for 24 h, embedded in paraffin sections and sectioned at 4 mm according to the standard procedure. Sections were deparaffinized, hydrated and then stained with hematoxylin and eosin (H&E). A single observer performed all the histological examination. The inflammatory changes in hearts were semi-quantitatively scored and graded based on severity of changes as follows: *Blood vessels*: − = Average, + = Mildly dilated/congested, + + = Moderately dilated/congested, + + + = Markedly dilated; *Edema:* − = No, + = Mild edema, + + = Moderate, + + + = Marked; *Hemorrhage:* − = No, + = Present.

### Statistical analysis

All variables were presented as means ± SEM. The one-sample Kolmogorov–Smirnov test was used to test for normality of variables. All variables were found to be normally distributed. One-way analysis of variance (ANOVA) was used to determine the differences between groups. In the case of a significant *F* value (*P* < 0.05), a least significant difference test was used to find significant intergroup differences. *P* values < 0.05 were considered statistically significant. All statistical data and statistical significance were performed by using SPSS statistical package (SPSS Inc.) version 20.0.1.

## Results

### Anthropometric measures

The initial and final body weight (BW), body mass index (BMI), and waist circumference (WC), all were significantly higher in old group compared to adult rats. Also, the percentage change in body weight (% BW) was significantly lower in old group compared to adult group. IF significantly decreased the % BW and % BMI in Old-F-ISO group compared to both old and Old-ISO groups, becoming significantly lower than those of adult group. Visceral adipose tissue weight (VATW) and visceral adipose tissue weight/body weight (VATW/BW) were not significantly different between old and adult groups. However, both were significantly decreased by IF in Old-F-ISO group in comparison with old, and Old-ISO groups, becoming even significantly lower than those of adult values (Table 1).

**Table 1:** Anthropometric measures, visceral fat, glucose homeostasis parameters and pancreatic Atg-7 in the four study groups

	Adult (10)	Old (6)	Old-ISO (7)	Old-F-ISO (7)
BW_i_ (g)	198.50 ± 7.95	366.66 ± 15.95^a^	353.57 ± 17.75^a^	376.42 ± 16.57^a^
BW_f_ (g)	217 ± 16.07	344.16 ± 19.51^a^	331.42 ± 20.16^a^	295 ± 14.67^a^
% BW (%)	8.85 ± 5.21	− 6.30 ± 2.40^a^	− 6.21 ± 3.35^a^	− 20.77 ± 5.26^a,b.c^
BMI_i_ (g/cm^2^)	0.50 ± 0.02	0.62 ± 0.03^a^	0.61 ± 0.03^a^	0.67 ± 0.02^a,c^
BMI_f_ (g/cm^2^)	0.53 ± 0.02	0.63 ± 0.03^a^	0.62 ± 0.04^a^	0.57 ± 0.01
% BMI (%)	5.23 ± 3.13	0.48 ± 2.63	0.69 ± 3.33	− 15.44 ± 2.30^a,b,c^
WC_i_ (cm)	14.50 ± 0.26	18.33 ± 0.33^a^	18.57 ± 0.44^a^	18.07 ± 0.25^a^
WC_f_ (cm)	13.20 ± 0.48	16.25 ± 0.70^a^	16.64 ± 0.47^a^	15.07 ± 0.56^a^
% WC	− 8.93 ± 2.93	− 11.48 ± 2.77	− 9.99 ± 3.74	− 16.54 ± 3.21
VATW (mg)	2937.00 ± 414.72	3311.67 ± 719.35	3414.29 ± 794.71	275.71 ± 184.62^a,b,c^
VATW/BW(mg/gm)	13.27 ± 1.00	9.48 ± 1.88	10.05 ± 2.28	0.87 ± 0.59^a,b,c^

Data are expressed as mean ± SEM

Number in parenthesis is the number of rats in each group

i, initial; f, final; % percentage change; BW, body weight; BMI, body mass index; WC, waist circumference; VTW, visceral tissue weight; VTW/BW; visceral tissue weight/body weight; FPG, fasting plasma glucose; FI, fasting insulin; HOMA-IR, homeostatic model assessment of insulin resistance; HOMA-%B, homeostatic model assessment of beta cell function

^a^Significance of difference from adult group by LSD test at *P* < 0.05

^b^Significance of difference from old group by LSD test at *P* < 0.05

^c^Significance of difference from old-ISO group by LSD test at *P* < 0.05

### Cardiac parameters

**Fig. 1:**
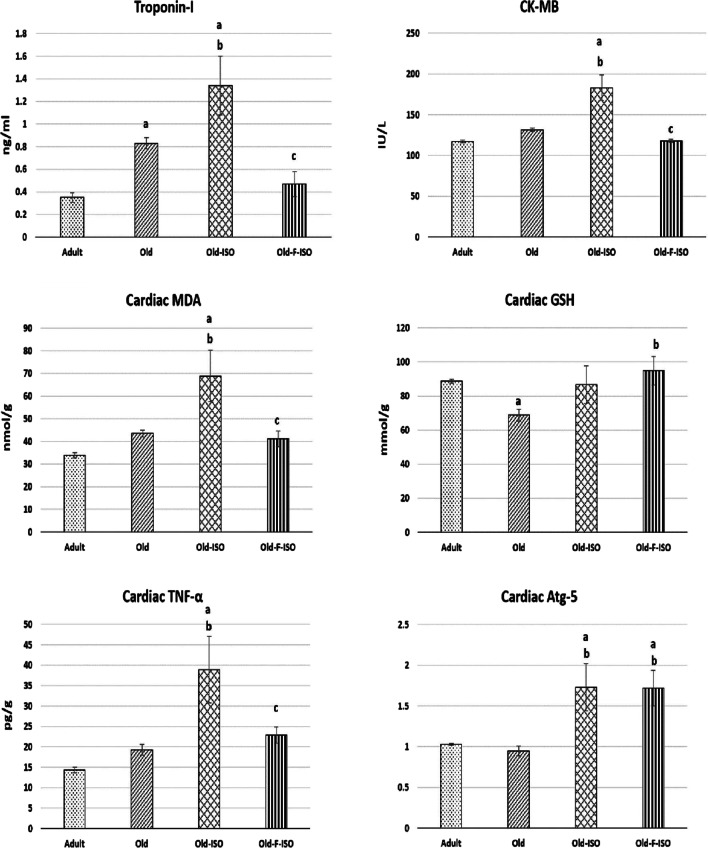
Changes in plasma level of Troponin-I, Creatine Kinase-MB (CK-MB), cardiac level of malondialdehyde (MDA), reduced glutathione reduced (GSH), tumor necrosis factor-alpha (TNF-α), and mRNA expression of cardiac autophagy related protein-5 (Atg-5) in the four study groups. Data are expressed as mean ± SEM. Number in parenthesis is the number of rats in each group. **a** Significance of difference from adult group by LSD test at *P* < 0.05. **b** Significance of difference from old group by LSD test at *P* < 0.05. **c** Significance of difference from Old-ISO group by LSD test at *P* < 0.05

As demonstrated in Fig. 1, the plasma level of cTnI was significantly higher in old group versus adult group. ISO administration significantly elevated troponin-I and CK-MB in Old-ISO group compared to both old and adult groups. IF in Old-F-ISO group significantly decreased both troponin-I and CK-MB versus Old-ISO group, approaching those of the adult values.

The cardiac level of GSH was significantly lower in old group versus adult group, while the cardiac levels of MDA and TNF-α, as well as the mRNA expression of the cardiac autophagy marker Atg-5, were not significantly different. ISO administration significantly elevated the MDA, TNF-α, and the mRNA expression of Atg-5 in Old-ISO group compared to both old and adult groups. IF in Old-F-ISO group significantly decreased the MDA and TNF-α in comparison with the Old-ISO group, reaching levels comparable to those of the adult group. Moreover, GSH showed a significant increase in Old-F-ISO compared to old group. Additionally, the mRNA expression of Atg-5 showed a significant higher level in Old-F-ISO group when compared to both old and adult groups, but it was not significantly different from Old-ISO group.

### Glucose homeostasis parameters and pancreatic autophagy marker

**Fig. 2:**
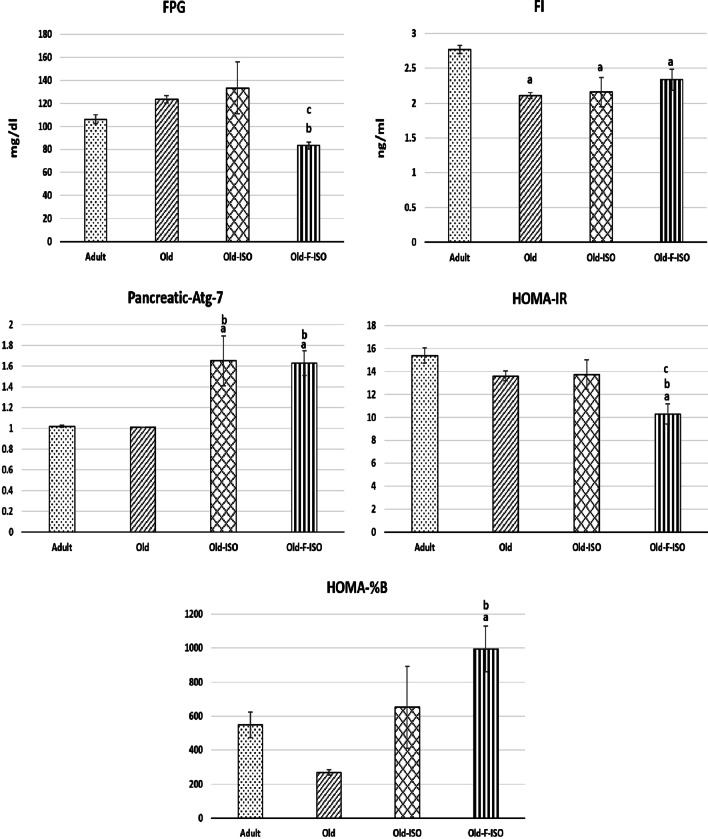
Changes in fasting plasma glucose (FPG), plasma level of fasting insulin (FI), mRNA expression of pancreatic autophagy marker (Atg-7), Homeostatic model assessment of insulin resistance (HOMA-IR), and Homeostatic model assessment of beta cell function (HOMA-%B), in the four study groups. Data are expressed as mean ± SEM. Number in parenthesis is the number of rats in each group. **a** Significance of difference from adult group by LSD test at *P* < 0.05. **b** Significance of difference from old group by LSD test at *P* < 0.05. **c** Significance of difference from Old-ISO group by LSD test at *P* < 0.05

Old rats presented a significantly lower FI associated with a higher FPG (though being insignificant), compared to adult group. The mRNA expression of pancreatic autophagy marker Atg-7, the HOMA-IR and HOMA-%B were not significantly different between old and adult groups (Fig. 2).

IF significantly decreased the FPG in Old-F-ISO versus old and Old-ISO groups. This was associated with a significant increase in the mRNA expression of pancreatic Atg-7 and HOMA-%B in Old-F-ISO group compared to old group, becoming significantly higher than that of the adult value. Also, IF significantly decreased HOMA-IR in Old-F-ISO compared to adult group.

ISO administration resulted in a significant increase in the mRNA expression of pancreatic Atg-7 compared to old group, becoming significantly higher than that of the adult value and approached those of Old-F-ISO groups.

### Histopathological results

**Fig. 3:**
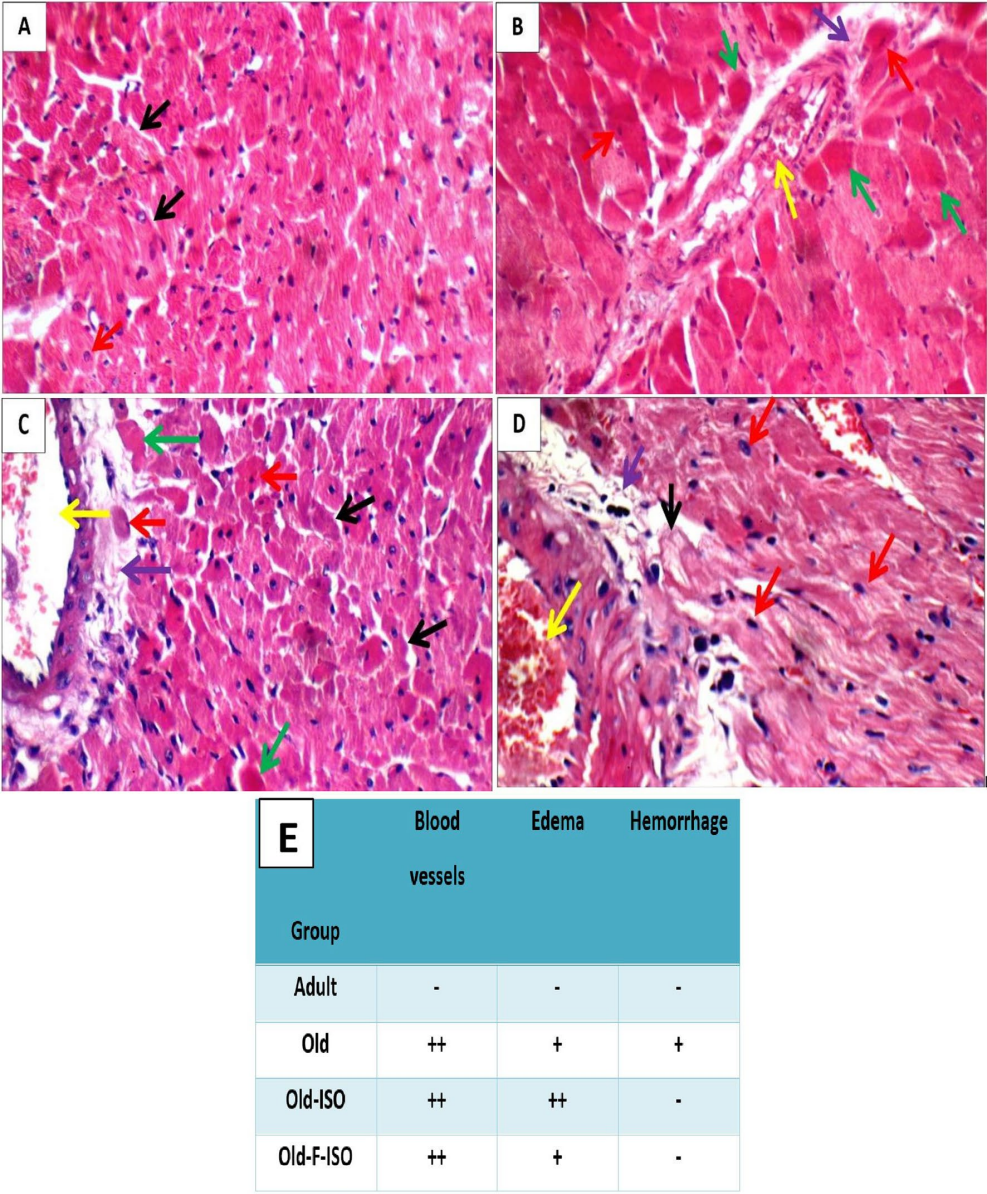
Photomicrograph of H&E-stained left ventricle sections of the four studied groups. **A** Adult group shows average muscle fibers with distinct cell borders (black arrows), and average centrally located nuclei (red arrow) (H&E X 400). **B** Old group shows most of cardiac muscle fibers with indistinct irregular cell borders and small pyknotic (red arrow) or no nuclei (green arrow), and mildly congested blood vessels (yellow arrow) with mild peri-vascular edema (purple arrow) (H&E X 400). **C** Old-ISO group shows few scattered cardiac muscle fibers with distinct irregular cell borders (black arrow), bright eosinophilic cytoplasm and small pyknotic (red arrow) or no nuclei (green arrow), and mildly dilated congested blood vessels (yellow arrow) with mild peri-vascular edema (purple arrow) (H&E X 400). **D** Old-F-ISO group shows viable cardiac muscle fibers with distinct regular cell borders (black arrow), and average nuclei (red arrow), and mildly dilated congested blood vessels (yellow arrow) with mild peri-vascular edema (purple arrow) (H&E X 400). **E** The histological inflammatory score

As demonstrated in Fig. 3, the left ventricles of the adult group showed average muscle fibers with distinct cell borders and average centrally located nuclei. Left ventricles of old group showed average pericardium, small areas of non-viable cardiac muscle fibers, and moderately congested blood vessels with mild peri-vascular edema. ISO administration moderately increases the histological inflammatory markers in Old-ISO rats. Hearts of Old-F-ISO group showed moderately dilated blood vessels and mild edema. Left ventricles of Old-F-ISO group showed viable cardiac muscle fibers with distinct regular cell borders, and average nuclei, and mildly dilated congested blood vessels with mild peri-vascular edema. The histopathological inflammatory score of different study group showed a mild to moderate inflammatory changes in left ventricles of old group versus adult group. IF attenuated the inflammatory markers in left ventricles of Old-F-ISO group.

## Discussion

Aging undermines cardiovascular homeostasis by enhancing slow and progressive functional and structural alterations of the heart, along with increased oxidative stress and inflammation; all of which promote the susceptibility to develop AMI [11, 24]. Old rats in the present study showed a significantly higher level of the troponin-I associated with a significantly lower level of the heart GSH compared to adult group, denoting cardiac tissue damage and diminished antioxidant defense mechanism in aged hearts. Also, histopathological examination of left ventricles of old group showed a mild to moderate inflammatory changes which could be explained by the decrease in the heart GSH and shift of oxidant/antioxidant balance toward oxidative stress. 

Decreased autophagy has been implicated in enhancing cardiovascular aging and increased liability to cardiovascular diseases [10, 25]. Impaired autophagic degradation of misfolded proteins produces mitochondrial injury, oxidative stress, increased inflammation, decreased ventricular contraction, and reduced tolerance to ischemic stress [26]. Old rats in the current work did not exhibit any significant change in mRNA expression of the cardiac autophagy marker, Atg-5, versus adult group. However, IF for 4 weeks significantly upregulated the cardiac autophagy, as evidenced by the significant increase in the mRNA expression of heart Atg-5 in Old-F-ISO group compared to old group, reaching levels significantly higher than those of adult values. Such chronic activation of the cardiac autophagy was associated with a significant decrease in serum levels of troponin-I, CK-MB in Old-F-ISO group compared to Old-ISO rats, approaching the levels of adult group. These results indicate that long-term activation of autophagy by IF could provide a potential cardioprotective effect against acute AMI in old rats. In line with these findings, Godar et al. demonstrated that IF protects against cardiac injury in mice (during ischemia/reperfusion) through increasing autophagic flux by stimulating nuclear translocation of TFEB, a master regulator of autophagy-lysosome gene expression networks [27].

Earlier studies have considered energy deprivation, oxidative stress and inflammation as serious events that take place during AMI and exaggerate cardiac injury. Lack of energy causes inhibition of Na^+^–K^+^ pump, increase in intracellular level of Ca^2+^, and mitochondrial dysfunction; resulting in activation of proteases, cleavage of anchoring cytoskeletal proteins, and progressive increase in cell membrane permeability with release of intracellular troponin-I and creatine kinase into the circulation [28, 29]. Moreover, mitochondrial damage during ischemia is critical incident, represents a key source of ROS that enhance oxidative cardiac injury [30, 31]. Also, recruitment of neutrophils and macrophages to the area of infarction activates the inflammatory reaction and promotes the oxidative stress, exaggerating the tissue damage [32, 33].

Autophagy is a naturally regulated process of lysosome-dependent turnover of damaged proteins and organelles that allows orderly degradation and recycling of cellular components [34]. It begins with the engulfment of lipid droplets, ribosomes, soluble proteins, or organelles in a double membrane autophagosomes; that when combined with lysosomes, continued enzymolysis occurs [35]. The products of autophagy, basic new nutrients, such as lipids, amino acids, and sugars, are then transported into the cytoplasm, where they are used as a source of energy [36]. It is activated by starvation or metabolic stress for the maintenance of tissue functions and homeostasis [37]. Moreover, mitophagy, selective mitochondrial autophagy, is the only intracellular degradative mechanism for removing damaged mitochondria and their harmful ROS [38, 39]. Also, autophagy was found to have an anti-inflammatory effect that attenuates the progression of cardiac damage [10, 25]. Hence, enhanced cardiac autophagy in Old-F-ISO group, in the current work, might provide a cardioprotective effect in response to ISO administration possibly by ensuring adequate nutrient supply under the circumstances of energy depletion, alleviating the local inflammatory reaction and by decreasing the cardiac oxidative tissue damage, perhaps via removing the damaged mitochondria, a fundamental source of ROS. In line with this assumption, the cardiac levels of MDA and TNF-α were significantly decreased in Old-F-ISO compared to Old-ISO group, in concomitance with increased expression of heart Atg-5. Additionally, Old-F-ISO group exhibited a significantly higher heart levels of the antioxidant enzyme, GSH, compared to old group. Moreover, histopathological studies, herein, revealed that IF can alleviate the local inflammatory response in Old-F-ISO versus Old-ISO group as manifested histologically by the attenuation of edema. 

Furthermore, attenuation of autophagy in beta cells of pancreas was recorded during aging and was assumed to induce age-related diabetic changes such as decreased insulin secretion, decreased beta cell mass and function, and hyperglycemia [40, 41]; well-known risk factors for ischemic heart disease [42]. Diabetes mellitus is known to proceed in stages characterized by alteration in blood glucose level, decrease in beta cell mass and function which progress gradually to significant hyperglycemia and frank diabetes with ketosis [43]. Although old rats in the present study did not present full picture of frank diabetes mellitus, they exhibited some criteria of age-related diabetic changes, manifested as a significant decrease in serum insulin level compared to adult group, associated with high FPG and low HOMA-B%. IF significantly upregulated the mRNA expression of Atg-7 in the pancreas of Old-F-ISO group versus old rats, reaching a level significantly higher than adult values, reflecting an increase in pancreatic autophagy. This was associated with a significant decrease in FPG in Old-F-ISO group when compared to old or Old-ISO groups. Also, enhancement of beta cell function was evident by IF, manifested as a significant increase in HOMA-B% in Old-F-ISO compared to old group, reaching a level significantly higher than that of adult rats. These observations suggest improved glycemic control by IF which might be induced by activation of pancreatic autophagy.

Autophagy has a fundamental homeostatic role necessary to maintain the structure, mass and function of pancreatic beta cells [44]. Hyperglycemia was reported to produce endoplasmic reticulum stress in β-cells [45], which enhances intracellular accumulation of misfolded proteins and promotes apoptosis [46]. Autophagy was found to have a role in the removal of harmful misfolded protein aggregates by directing cytosolic contents to the lysosome for degradation [47].

Hyperglycemia is a serious risk factor for coronary heart disease and is strongly related to the high mortality rate in patients with acute MI [48]. Previous studies reported that hyperglycemia can produce a direct damaging effect on ischemic myocardium by reducing collateral circulation, increasing infarct size [49], and enhancing apoptosis [50]. Controlling hyperglycemia was found to produce a significant reduction in the morbidity and mortality of acute AMI patient [51]. Therefore, upregulation of the pancreatic autophagy by IF, in the present study, might assign an additional protective mechanism against acute AMI induced by ISO via amelioration of the hyperglycemia in old rats.

The present non-significant changes in heart and pancreatic autophagy in old group versus adult rats, despite the presence of cardiac injury as well as diabetic changes, notify that age-related cardiovascular and metabolic alteration could be mediated by other risk factors rather than altered autophagy. In the present study, an obvious increase in obesity markers (significant increase in final BW, BMI and WC) was significantly recorded in old versus adult group. IF decreased % BW, % BMI, VATW and VATW/BW in Old-F-ISO group when compared to old, Old-ISO and adult groups. These findings indicate a decrease in overall and visceral obesity by IF in old rats.

The association between obesity and aging has been demonstrated by previous studies [52, 53]. Obesity, particularly visceral obesity, is the cornerstone for metabolic disorders which provokes the onset of insulin resistance, diabetes mellitus, hypertension, and dyslipidemia [54, 55], imposing a great burden on the cardiovascular system, and increasing the susceptibility to AMI during aging [56]. IF diet regimens were found to reduce the risk of obesity both in animal [57] and human studies [58]. IF was found to improve indicators of coronary heart disease in obese men and women, such as reducing body weight, waist circumference, and body fat mass [59]. Previous reports have recorded a cardioprotective impact of IF diet and attributed such effect to the reduction in fat tissue, especially visceral fat tissue [60, 61]. Moreover, IF increases the utilization of fat, directing the body metabolism toward a ketogenic state that increases weight loss, as processing ketones consumes high energy [62]. Therefore, attenuation of the age-related obesity, herein, represents another cardioprotective mechanism of IF, alleviating the severity of AMI.

Importantly, Old-ISO group, in the present study, presented a significantly higher levels of cardiac injury markers (Troponin-I and CK-MB); cardiac levels of oxidative stress marker (MDA) and proinflammatory marker (TNF-α) in association with a significant increase in mRNA expression of heart Atg-5 versus old and adult groups. Similarly, pancreatic autophagy marker Atg-7 was significantly increased in Old-ISO group in comparison with both old and adult groups. Of note, autophagy, together with apoptosis and necrosis, plays a dichotomous ‘survival and death’ role in cell homeostasis [63]. Despite, it is activated in ischemic stress in attempt to conserve cardiomyocyte in face of ischemic injury [64], excessive autophagy may be harmful by increasing cell death, a process called autosis [55]. Moreover, excessive activation of autophagy may lead to a detrimental effect on the heart during cardiac ischemia, as well as during the reperfusion stage [65]. Additionally, overstimulation of autophagy was found to impair beta cell function in vitro and in vivo studies [66]. Unregulated autophagy can be deleterious and result in autophagic cell death [67]. Therefore, the current cardiac oxidative stress, inflammation, and tissue damage in association with upregulation of Atg-5, observed in Old-ISO group, might be explained by the acute stimulation of autophagy during acute cardiac ischemia. Likewise, the pancreatic stress induced by ischemic changes during acute AMI in Old-ISO group sharply overstimulated the pancreatic autophagy which might damage beta cells, particularly, these rats presented a significantly high FPG compared to Old-F-ISO group. On the other hand, long-term activation of both heart and pancreatic autophagy markers by IF, herein, afford a protective mechanisms against acute cardiac insult in Old-F-ISO group.

## Conclusions

IF increases the tolerance of aged myocardium to acute cardiac insult induced by ISO administration in old rats, possibly by activation of both cardiac and pancreatic autophagy, ensuring sufficient energy supply to the heart, decreasing the cardiac oxidative stress and inflammation, and alleviating the age-related diabetic changes. Also, amelioration of age-related overall and visceral obesity could be an additional mechanism of IF, which afford protection against AMI. Hence, IF could be a dietary lifestyle modification for attenuation of the susceptibility to acute AMI in aged population. Moreover, this study indicates the controversial effect of autophagy, providing evidence that chronic rather than acute activation of both cardiac and pancreatic autophagy may potentially confer such cardioprotective effect against AMI. Future studies are required to further investigate the difference between acute and chronic activation of autophagy and their effects on different body systems in elderly.

## Data Availability

Our data are available (on figshare) for share upon reasonable request.

## Abbreviations

AMI: Acute Myocardial infarction
IF: Intermittent fasting
ISO: Isoproterenol
Old-ISO: Old-isoproterenol group
Old-F-ISO: Old-intermittent fasting-isoproterenol group
FPG: Fasting plasma glucose
FI: Fasting insulin
CK-MB: Creatine kinase-myocardial band
cTnI: Troponin-I
MDA: Malondialdehyde
GSH: Reduced glutathione
TNF-α: Tumor Necrosis factor-alpha
Atg-5: Autophagy related-5
Atg-7: Autophagy related-7
HOMA-IR: Homeostasis Model Assessment of Insulin Resistance
HOMA-%B: Homeostasis Model Assessment of Beta Cell Function
VATW: Visceral adipose tissue weight
VATW/BW: Visceral adipose tissue weight/body weight
